# Impact of arsenic exposure on the gut-microbiota-liver axis in poultry

**DOI:** 10.1016/j.psj.2026.108078

**Published:** 2026-11-03

**Authors:** Xinying Zhou, Fuhao Chen, Qingjia Lin, Xinyu Zhang, Ting Zhao, Falong Yang, Kai Zhang, Haifeng Liu, Shiwen Xu, Dechun Chen

**Affiliations:** a Key Laboratory of Animal Medicine of Sichuan Education Department, Southwest Minzu University, Chengdu, 610041, China; b Chongqing Fengdu Agricultural Science and Technology Development Group Co., Ltd. Chongqing, 408200, China; c Department of Veterinary Surgery, College of Veterinary Medicine, Sichuan Agricultural University, Chengdu, 611130, China; d College of Veterinary Medicine, Northeast Agricultural University, Harbin, 150030, China

**Keywords:** Arsenic, Poultry, Gut-microbiota-liver axis, Intestinal barrier, Oxidative stress

## Abstract

Arsenic (As), a priority hazardous substance widely distributed in nature, endangers poultry health and food safety. The gut‑microbiota‑liver axis forms a bidirectional regulatory network, with the intestine acting as the main absorption site and the liver as the key metabolic‑detoxification organ for As. This review summarizes arsenic‑induced toxic injuries to this axis in poultry. In the intestine, As impairs mucosal barrier function, triggers epithelial cell death via NF‑κB‑NLRP3 inflammasome activation, and causes leaky‑gut syndrome. It also disturbs gut microbiota homeostasis, lowering beneficial butyrate‑producing bacteria and impairing short‑chain‑fatty‑acid synthesis. In the liver, As disrupts energy metabolism, provokes severe oxidative stress, and triggers multiple forms of programmed cell death, including ferroptosis and PANoptosis. Gut-derived lipopolysaccharide translocates to the liver via the portal circulation, amplifying hepatic inflammation, which in turn causes altered bile acid metabolism and reduced short-chain fatty acid production, thereby triggering gut microbiota dysbiosis and forming a self-reinforcing vicious cycle. Potential interventions including probiotics, polysaccharides, selenium and vitamin E are discussed to counteract arsenic toxicity. This work provides theoretical support for arsenic risk control in poultry farming.

## Highlights

• Arsenic induces intestinal barrier damage and gut microbiota dysbiosis in poultry.
• Gut‑liver axis dysfunction mediates arsenic‑provoked hepatic injury in poultry.
• Arsenic‑triggered hepatotoxicity involves ferroptosis and other cell‑death modes.
• Arsenic is identified as a gut‑liver‑axis toxicant in poultry toxicology.

## Graphical abstract

**Figure:**
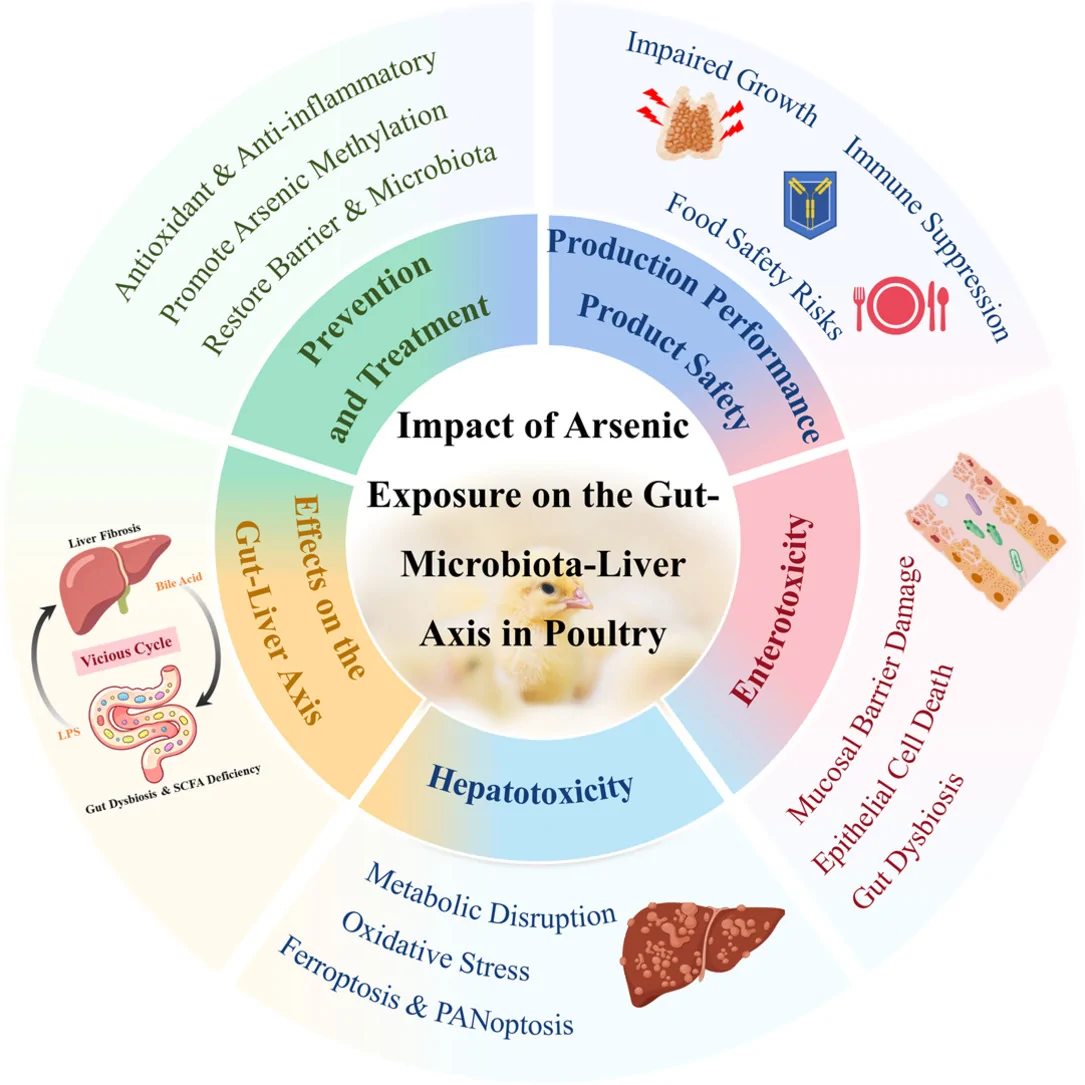

## Introduction

Arsenic (As) is a metalloid element widely distributed in nature, and its continuous contamination of water, soil, and air through geological activities, industrial emissions, and the use of arsenic‑containing pesticides has led to its designation as a priority hazardous substance by the U.S. Agency for Toxic Substances and Disease Registry (ATSDR) (ATSDR, 2009; Faria et al., 2023). The toxicity of As is closely related to its chemical speciation: it is generally accepted that inorganic arsenic species are more toxic than organic ones, with inorganic trivalent arsenic (iAs^III^) and inorganic pentavalent arsenic (iAs^V^) being the most potent, and both are classified as Group I human carcinogens by the International Agency for Research on Cancer (IARC) (IARC, 2012).

Poultry constitutes a core component of the global livestock industry and represents a primary source of animal‑derived protein for human consumption (Kim et al., 2019). In poultry production, it has been proposed that very low doses of arsenic (0.012-0.050 mg/kg) may participate in methionine metabolism (Živkov Baloš et al., 2019). However, this conclusion is primarily based on observations under specific experimental conditions. The NRC (2005) does not consider arsenic to be a universally essential nutrient, and arsenic is also teratogenic and carcinogenic (National Research Council, 2005); therefore, its “essentiality” remains controversial. In practical production, arsenic exposure often results from the misuse of organic arsenic additives as growth promoters; in addition, common exposure routes include contaminated soil and water, as well as arsenic-containing metabolic transformation products in poultry manure (Arai et al., 2003; Missimer et al., 2018; Yang et al., 2016). Excessive arsenic exposure leads to accumulation in poultry tissues and organs, resulting in decreased body weight, reduced feed conversion ratio, and increased mortality under the multifaceted effects of arsenic toxicity (Chen and Costa, 2021; Roy et al., 2013). This directly undermines the economic benefits for producers and increases the risk of arsenic residues entering the human dietary chain through meat and egg products, posing a potential public health threat (Aljohani, 2023; Wei et al., 2021). Currently, most countries have established strict maximum limits for inorganic arsenic in poultry meat and products, and have even banned the use of common feed additives such as roxarsone (ROX).

The intestine is the primary site of arsenic absorption, and both its physical and biological barriers are significantly damaged under arsenic exposure (Palma-Lara et al., 2020; Zhong et al., 2021). The liver, as the core organ for arsenic metabolism and detoxification, highly expresses arsenic methyltransferase (As3MT) and is highly susceptible to arsenic insult, manifesting as steatosis, fibrosis, and even carcinogenesis. These two organs, in turn, form a close bidirectional regulatory network via the gut-microbiota-liver axis (Li et al., 2025).

The gut-microbiota-liver axis is a bidirectional regulatory system formed by the intestine, liver, and gut microbiota through anatomical structures, biochemical metabolism, and immune signals, capable of integrating multiple interactions among organs, microbiota, diet, and the environment (Albillos et al., 2020; Tripathi et al., 2018). Its core pathways are the portal vein circulation and bile acid secretion: metabolites of the gut microbiota (e.g., lipopolysaccharide, LPS) can enter the liver via the portal vein, regulating hepatic immune and metabolic functions; conversely, bile acids synthesized by the liver, upon entering the intestine, can also modulate the microbiota and barrier functions (Rajapakse et al., 2023).

Arsenic exposure can disrupt the homeostasis of this axis, forming a vicious cycle that amplifies toxicity. Specifically, arsenic exposure reduces the abundance of beneficial bacteria in the chicken ileum, such as *Christensenellaceae* and *Ruminococcaceae*, damages the intestinal barrier, and promotes the translocation of gut‑derived LPS to the liver; LPS subsequently activates the TLR4/NF‑κB pathway in hepatocytes, facilitating the release of pro‑inflammatory cytokines and exacerbating liver injury (Li et al., 2025). Concurrently, the expression of CYP7A1, the rate‑limiting enzyme for hepatic bile acid synthesis, is upregulated, and the excessive bile acids refluxing into the intestine further aggravate intestinal damage and dysbiosis (Wang et al., 2024).

Current research on arsenic toxicity in poultry remains predominantly focused on single organs, lacking a systematic synthesis of the microecological interactions among the intestine, liver, and microbiota; moreover, poultry-specific mechanistic evidence is limited, dose-time-effect relationships remain poorly defined, and comparative toxicity data across arsenic species are still scarce. This review summarizes recent relevant studies, systematically analyzes the toxic effects of arsenic on the intestine and liver in poultry, and, taking the gut-microbiota-liver axis as the central framework, correlates arsenic‑induced liver injury, microbial dysbiosis, and growth inhibition, elucidating the underlying causal and amplification mechanisms. This provides a theoretical basis for screening molecular intervention targets, developing prevention and control strategies, and safeguarding poultry health and food safety.

## Effects of arsenic exposure on poultry production performance and outcomes

### Impairment of production performance by arsenic exposure

When dietary arsenic content reaches 40-50 mg/kg, production performance is markedly impaired: 40 mg/kg causes a decline in egg production, and 50 mg/kg leads to a reduced feed conversion ratio, accompanied by decreased body weight and increased mortality, severely compromising the economic benefits for producers (Živkov Baloš et al., 2019). Arsenic impedes growth primarily by inducing oxidative stress, impairing liver and kidney function, and inhibiting nutrient absorption. Furthermore, arsenic can accumulate in organs such as the heart, resulting in developmental abnormalities; for example, in white‑feathered ducks exposed to arsenic, both the absolute and relative heart weights are significantly reduced (Kumar Panda et al., 2023; PIZARRO et al., 2015).

Long‑term exposure to an arsenic‑contaminated environment directly suppresses the immune capacity of poultry: first, arsenic accumulates in the bursa of Fabricius, causing its atrophy, hindering lymphocyte differentiation and maturation, and reducing the synergistic effect of T and B lymphocytes (Subhashree et al., 2020); second, it activates the cGAS-STING pathway, a key innate immune signalling pathway in cells, and STING can further activate the NF‑κB pathway, leading to exacerbated inflammation and impaired immunoregulatory function (Pan et al., 2024); and third, it induces oxidative stress, with accumulated reactive oxygen species (ROS) prompting cells to release pro‑apoptotic factors such as cytochrome c, which activate the caspase cascade and induce lymphocyte apoptosis (Liu, 2025; Sattar et al., 2016). Under the combined influence of the above factors, poultry ultimately exhibit diminished innate and adaptive immunity, reduced vaccine efficacy, increased susceptibility to pathogenic bacteria, and significantly impaired production performance (Ren, Deng, et al., 2021; Sattar et al., 2016).

### Safety concerns of poultry products following arsenic exposure

Poultry meat products and eggs are important sources of animal protein in the human diet. However, poultry have a high bioaccumulation capacity for arsenic, which can be detected in muscle tissues as well as in visceral organs such as the liver, kidney, and heart, and the hazard is magnified through the food chain, posing a potential health risk (Nachman et al., 2013; Sun, 2014). On the other hand, ROX is commonly used in production as a growth‑promoting additive, which elevates the total arsenic level in poultry manure, the subsequent land application of manure leads to secondary environmental contamination (Christen, 2006; Stolz et al., 2007). Currently, many countries and regions, including the United States and Europe, have established strict maximum limits for inorganic arsenic in poultry meat and products, and are progressively restricting the use of organic arsenic feed additives such as ROX (Chapman and Rathinam, 2022).

## Intestinal toxicity of arsenic in poultry

Following ingestion through feed and drinking water, approximately 80% of arsenic can be absorbed by the intestine, subsequently damaging the intestinal mucosal barrier and disrupting gut microbiota homeostasis (Palma-Lara et al., 2020). Currently, mechanistic research on arsenic‑induced intestinal toxicity in poultry remains relatively scarce. This section summarizes the available evidence from poultry studies, while also referring to shared injury pathways identified in other species, to systematically delineate the intestinal toxic effects of arsenic in poultry.

### Arsenic-induced damage to the intestinal mucosal barrier in poultry

Arsenic damages the intestinal mucosal barrier in poultry mainly by injuring intestinal epithelial cells and downregulating multiple key molecules that maintain intestinal integrity (Turner, 2009). Macroscopically, this manifests as extensive atrophy, breakage, or even complete shedding of small intestinal villi, along with damage and loss of crypt structures, leading to a severe reduction in the effective absorptive surface area of the intestine and impaired nutrient absorption (Chen et al., 2025b). Arsenic affects the survival and repair of intestinal epithelial cells primarily through two pathways: first, it directly damages intestinal epithelial cells by upregulating the phosphorylation and nuclear translocation of p65 protein in the NF‑κB pathway, thereby reducing the regenerative capacity of the intestinal mucosa (Jeong et al., 2017); second, it induces programmed cell death in intestinal epithelial cells, particularly by activating Caspase‑1‑mediated pyroptosis and Caspase‑3‑mediated apoptosis, which result in exacerbated cell death within the intestinal barrier (Zhong et al., 2021).

In poultry, arsenic exposure downregulates intestinal barrier-related proteins, including the tight junction proteins ZO-1, Occludin, and Claudin-1, as well as the mucin MUC2, leading to increased intestinal permeability and elevated LPS levels (Ibrahim et al., 2022; Zhong et al., 2021). Mechanistic evidence from mammalian intestinal epithelial cells indicates that arsenic-induced downregulation of claudin proteins is mediated through oxidative stress-dependent activation of p38 MAPK and NF-κB p65(Yahong and Weihua, 2025); however, direct evidence confirming the operation of this p38/NF-κB-tight junction axis in the poultry intestine remains limited. The reduced transcription and protein expression of these molecules lead to widened intercellular spaces in the intestinal epithelium, abnormally increased intestinal permeability, and the development of a leaky gut (Ibrahim et al., 2022). After disruption of the intestinal barrier, endotoxins such as LPS released by Gram‑negative bacteria undergo translocation. Experimental evidence indicates that LPS activates Toll‑like receptor 4 (TLR4) on epithelial cells and lamina propria immune cells, subsequently triggering the downstream NF‑κB signalling pathway, which potently promotes the transcription and release of pro‑inflammatory cytokines such as TNF‑α, IL‑6, and IL‑1β. Concurrently, damage‑associated molecular patterns (DAMPs) elicited by arsenic can activate the NLRP3 inflammasome, leading to Caspase‑1 activation, which further processes and releases mature IL‑1β and IL‑18, thereby exacerbating the inflammatory cascade (Jeong et al., 2017; Turner, 2009).

### Arsenic disruption of the gut microbiota

Arsenic alters the composition and metabolic functions of the gut microbiota in poultry, and the resulting dysbiosis further interacts with intestinal mucosal injury and hepatic inflammation, exacerbating systemic damage.

At the community structure level, arsenic disrupts gut microbial homeostasis. In layer hen models, arsenic treatment significantly decreased alpha diversity (Shannon, Faith PD indices) and shifted beta diversity, indicating a specific alteration in overall microbial composition (Li et al., 2025; Y. Yang et al., 2023). At the phylum level, this layer hen study showed that arsenic treatment significantly increased the *Firmicutes/Bacteroidetes* ratio while reducing the combined relative abundance of beneficial bacteria such as *Christensenellaceae* and *Ruminococcaceae* (Li et al., 2025; Liu et al., 2025). Additional studies have reported that arsenic exposure alters the gut microbiota composition in poultry, characterized by enrichment of *Bacteroidetes* and depletion of *Firmicutes* (McDermott et al., 2020; Zhou et al., 2025). At the genus level, the abundance of arsenic-sensitive probiotics (e.g., *Lactobacillus*) decreased (Zhou et al., 2025). It should be noted that the arsenic exposure models, doses, and exposure durations vary greatly among studies, and the direction of microbiota responses is not always consistent. For example, in mouse models, arsenic decreased the relative abundance of hydrogen sulfide-producing *Desulfovibrio* and reduced intestinal H₂S levels, rather than enriching them (Chen et al., 2025b); these results cannot be directly compared with poultry data. Therefore, species, dose, exposure duration, and sequencing methods should all be considered when interpreting the effects of arsenic on the gut microbiota.

At the functional level, the metabolic products of the gut microbiota are altered compared with those in healthy individuals, directly or indirectly exacerbating intestinal barrier injury, systemic inflammation, and metabolic disturbances in poultry. First, the synthesis of beneficial metabolites such as short‑chain fatty acids (SCFAs), including butyrate and acetate, may be impaired owing to a reduction in acid‑producing bacteria, thereby weakening their roles in providing energy for intestinal epithelial cells, exerting anti‑inflammatory effects, and maintaining barrier integrity (Rajapakse et al., 2023). Second, arsenic disrupts the normal function of the farnesoid X receptor (FXR): when bile acids are excessive, FXR can inhibit bile acid synthesis and promote their intestinal excretion, but upon FXR inhibition, bile acid metabolism becomes dysregulated, and Th17 cell differentiation is promoted, exacerbating intestinal inflammation (Y. Yang et al., 2023; Zhao et al., 2025). More critically, the gut microbiota itself is an important participant in arsenic biotransformation, influencing the speciation, solubility, and toxicity of arsenic through methylation, reduction, and other reactions (Kezer et al., 2025). Specific genera such as *Clostridium* and *Desulfovibrio* have been shown to mediate these transformations, though direct evidence in poultry remains scarce. Arsenic poisoning perturbs this transformation process, altering the existing forms of arsenic in the intestine (e.g., converting methylated products into more toxic inorganic arsenic), and facilitating translocation through intestinal epithelial cells into the circulatory system, thereby affecting the efficiency of intestinal arsenic absorption, tissue distribution, and overall toxic effects (Roggenbeck et al., 2019).

## Hepatotoxicity of arsenic in poultry

Environmental arsenic enters poultry mainly through feed and drinking water, and the liver is the most important organ for detoxification and metabolism, as well as the primary site for arsenic accumulation and speciation transformation (Li et al., 2025). In the poultry liver, the arsenic methylation pathway follows the generally conserved scheme: ingested As^V^ is first reduced to As^III^ by glutathione (GSH); subsequently, AS3MT, using S‑adenosylmethionine (SAM) as a methyl donor, catalyses the methylation of As^III^ to monomethylarsonic acid (MMA), which is further methylated to the less toxic dimethylarsinic acid (DMA) (Peng et al., 2017; Roy et al., 2013). The basic catalytic requirement for SAM and reduced thiols appears conserved across vertebrates. However, direct comparative data on whether the expression level, substrate affinity, or catalytic efficiency of hepatic AS3MT in poultry differs from that in mammals remain limited, and the consequences of any such differences for arsenic metabolism and toxicity in poultry are not well defined. A large number of studies have shown that arsenic exposure increases the probability of hepatic steatosis, fibrosis, and even tumorigenesis (Wei et al., 2021; Ren, Deng, et al., 2021) demonstrated that dietary supplementation with 3 mg/kg arsenic induced hepatic oxidative stress and steatosis in poultry, inhibited growth, and caused economic losses in production.

### Arsenic interference with energy metabolism and cellular metabolism in poultry hepatocytes

Arsenic entering the body predominantly exists in trivalent and pentavalent organic forms, which mainly affect hepatocyte energy metabolism and cellular metabolism by interfering with ATP synthesis and inhibiting key enzyme activities (Hughes, 2002). Among these, As^V^, owing to its structural similarity to phosphate, can substitute for phosphate in biochemical reactions such as glycolysis, forming unstable arsenate esters (e.g., glucose‑6‑arsenate) that prevent the formation of high‑energy phosphate bonds, thereby blocking ATP production. For example, in the reaction catalysed by glyceraldehyde‑3‑phosphate dehydrogenase (GAPDH), As^V^ combines with glyceraldehyde‑3‑phosphate to generate 1‑arseno‑3‑phosphoglycerate, which is unstable and readily hydrolyses spontaneously, hindering substrate‑level phosphorylation and ultimately resulting in reduced ATP synthesis (Ren, Deng, et al., 2021). However, current evidence supporting this glycolytic interference mechanism is mainly derived from in vitro and mammalian studies, and direct evidence in poultry hepatocytes remains scarce; this is also one of the key directions for future research.

As and its methylated metabolic intermediates (e.g., MMA) readily bind to thiol groups (-SH) at the active centres of enzymes, inhibiting the activity of multiple key enzymes and disrupting cellular metabolism. Typical examples include pyruvate dehydrogenase and glutathione reductase; in addition, studies have observed that As can bind to the selenocysteine (Sec) residue in glutathione peroxidase (GSH-Px), inhibiting its catalytic activity and thereby reducing GSH-Px activity in broiler hepatocytes, which weakens cellular antioxidant defence capacity (Packianathan et al., 2018). Similar cases have been reported by Roggenbeck et al. (Roggenbeck et al., 2019), where As binds to thioredoxin reductase (TrxR) and other thiol‑dependent antioxidant enzymes, further exacerbating redox imbalance. AS3MT, a key enzyme in arsenic biotransformation, contains cysteine residues (Cys44/Cys174/Cys224) that are essential components of its active centre and critical binding sites for As. When the concentration of As in the system is excessively high, the surplus As persistently occupies the enzyme’s active sites and can also oxidatively modify the cysteine residues, disrupting the AS3MT active centre and preventing the enzyme from properly binding subsequent substrates (e.g., MMA) or the methyl donor (SAM), ultimately leading to accumulation of As in vivo (Packianathan et al., 2018; Zhong et al., 2021).

### Arsenic-induced oxidative stress and antioxidant system imbalance in poultry hepatocytes

Arsenic induces oxidative stress in hepatocytes through multiple synergistic pathways, which can be briefly summarized as excessive production of reactive oxygen species (ROS) and depletion of the antioxidant defense system. On one hand, the excessive ROS production involves the following processes: arsenic activates the cGAS‑STING and NF‑κB pathways, promoting the secretion of inflammatory cytokines such as TNF‑α and IL‑6, recruiting neutrophils and releasing myeloperoxidase (MPO), which in turn promotes ROS production (Pan et al., 2024; Ren, Deng, et al., 2021); simultaneously, arsenic can inhibit mitochondrial respiratory chain complex function, leading to electron leakage and the generation of superoxide anion (O₂⁻) and hydrogen peroxide (H₂O₂), interfering with the mitochondrial electron transport chain and impairing mitochondrial function (Prakash et al., 2022; Roggenbeck et al., 2016); moreover, arsenic can activate cytochrome P450 enzymes (e.g., CYP450) and xanthine oxidase (XO), catalyzing ROS generation, and also inhibit the NER/BER repair pathways (for repairing DNA damage) and interfere with DNA ligase activity, resulting in lipid peroxidation and DNA damage (Su et al., 2023; Zhong et al., 2022). On the other hand, the depletion of the antioxidant system is reflected in two aspects: GSH consumption and antioxidant enzyme inhibition. First, As binds to the ‑SH of GSH to form As‑GSH complexes, leading to rapid depletion of intracellular GSH and weakening its free radical scavenging capacity (Roy et al., 2013). Second, arsenic can directly bind to the active sites of superoxide dismutase (SOD) and GSH‑Px (especially the Sec residue), inhibiting their catalytic activities and further reducing the cellular capacity to scavenge ROS (Li et al., 2025). These multiple ROS-producing pathways do not act in isolation but form a synergistic network.

### Arsenic toxicity activates the ferroptosis pathway in poultry hepatocytes

On the basis of metabolic disturbance and oxidative stress, arsenic further disrupts iron homeostasis, induces mitochondrial damage, and activates programmed death pathways, leading to ferroptosis and other forms of cell death in hepatocytes. Arsenic inhibits the Nrf2 signaling pathway, thereby downregulating the expression of ferroportin (FPN) and heme oxygenase‑1 (HO‑1), reducing cellular iron efflux and resulting in intracellular iron accumulation; subsequently, arsenic promotes the binding of ferritin to lysosomes and its degradation through NCOA4‑mediated ferritinophagy or the AMPK/mTOR pathway, releasing free iron (Li et al., 2025; Ren, Deng, et al., 2021; Roggenbeck et al., 2019). The iron ions then catalyze the accumulation of lipid peroxides and hydroxyl radicals via the Fenton reaction, ultimately triggering autophagy‑dependent ferroptosis.

In addition to inducing ferroptosis, arsenic exposure can simultaneously activate mitochondrial apoptosis and death receptor pathways. Recent studies have further revealed that arsenic upregulates the expression of sensor proteins such as ZBP1, which in turn integrate and synchronously trigger the key signaling pathways of apoptosis, pyroptosis, and necroptosis, forming a novel, irreversible pattern of cell death termed "PANoptosis" (Su et al., 2024). However, because the ZBP1-centered PANoptosis framework was originally established in mammals, whether the expression and regulatory features of ZBP1 in poultry fully match those in mammals remains to be verified, and its conservation in birds should be regarded as an assumption supported by phenotypic evidence. This mode is not an independent activation of a single pathway, but rather, through molecular platforms such as ZBP1, it concurrently activates Caspase‑8/7/3‑mediated apoptosis, NLRP3/ASC/GSDMD‑mediated pyroptosis, and RIPK1/RIPK3/MLKL‑mediated necroptosis, leading to extensive hepatocyte death (Chen et al., 2025a).

It should be noted that ferroptosis and PANoptosis are not completely independent. Studies have shown that oxidative stress and lipid peroxidation can simultaneously participate in both death programs, and in hypoxic cells, lipid peroxidation scavengers can simultaneously alleviate ferroptosis and PANoptosis, suggesting molecular crosstalk between the two (Sun et al., 2025). However, in arsenic-induced poultry hepatocyte injury, whether ferroptosis acts as an upstream signal to trigger PANoptosis, or whether ZBP1 directly integrates ferroptosis signals, currently lacks direct evidence. The upstream-downstream relationship between the two in arsenic poisoning models remains a research hotspot in this field and awaits further validation (Fig. 1).

**Figure 1:**
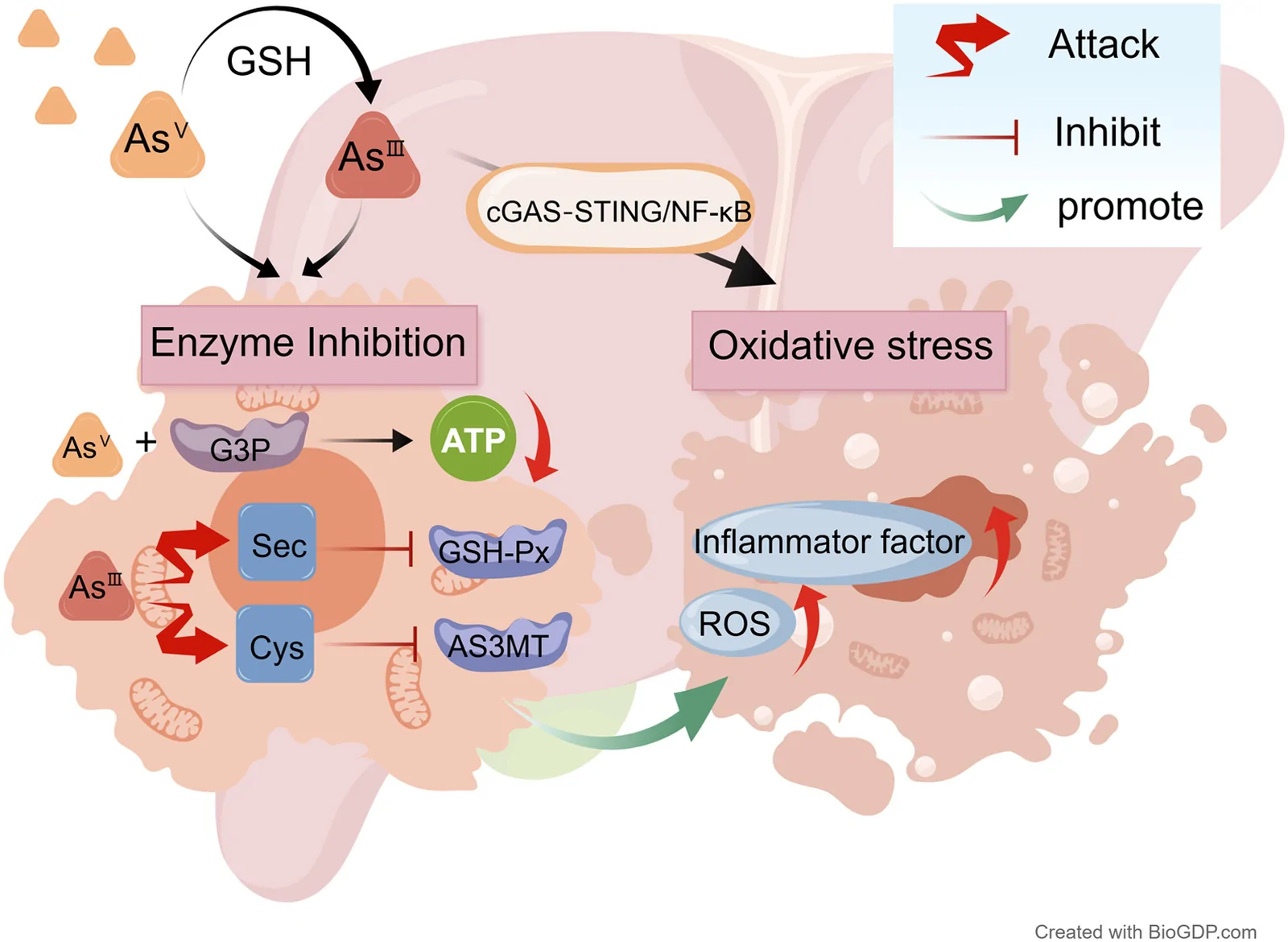
Arsenic triggers hepatocyte injury via enzyme‑inhibition‑mediated selenium metabolism disturbance, oxidative stress and cGAS‑STING/NF‑κB‑driven inflammatory responses.

## Effects of arsenic on the gut-microbiota-liver axis in poultry

### Bidirectional regulation of the gut-microbiota-liver axis

The intestine, gut microbiota, and liver in poultry rely on blood circulation to achieve bidirectional communication of metabolic and immune signals, and these three components form a dynamic regulatory network through two major pathways: the bile acid circulation and the immune regulation by gut metabolites (Li et al., 2025). At the metabolic level, the liver synthesises primary bile acids and secretes them into the intestine, where they are converted into secondary bile acids by bile salt hydrolase (BSH) from intestinal bacteria such as *Ruminococcaceae* and *Faecalibacterium* (Institute, 2024; Perino et al., 2021). The secondary bile acids return to the liver via the portal vein, activate hepatocyte FXR, and downregulate the key enzyme for bile acid synthesis, CYP7A1, thereby stabilising the total bile acid pool through negative feedback (Perino et al., 2021; Song et al., 2025); meanwhile, secondary bile acids can modulate gut microbiota composition and inhibit the proliferation of harmful bacteria (Zhao et al., 2025). At the immune level, short‑chain fatty acids (predominantly butyrate) generated by the gut microbiota serve as an important energy source for intestinal epithelial cells. Butyrate epigenetically upregulates tight junction proteins such as ZO‑1 and Occludin, thereby reinforcing the intestinal barrier (J. Yang et al., 2023). Under homeostatic conditions, a healthy microbiota reduces LPS release, maintaining low portal endotoxin levels and keeping hepatic Kupffer cells quiescent, thus preventing excessive inflammatory responses (Heymann et al., 2015).

### Arsenic disruption of gut-microbiota-liver axis homeostasis

Initially, after entering the body, arsenic interferes with the interactions among the intestine, microbiota, and liver from multiple aspects, disrupting the homeostasis of the gut-microbiota-liver axis and aggravating systemic damage in poultry (Zhong et al., 2021). During this process, both the intestine and liver are target organs of arsenic. The normal functions of these three pathways and their disruption by arsenic are systematically summarized in Table 1.

**Table 1:** Arsenic disruption of the three core pathways in the gut-microbiota-liver axis.

Pathway	LPS/Portal vein	Bile acids/FXR	SCFA/Butyrate
Normal Homeostasis	Low endotoxin levels Kupffer cell quiescence	Negative feedback inhibition of CYP7A1 Maintenance of bile acids pool homeostasis	Provides energy for intestinal epithelium Upregulates tight junction proteins
Arsenic Interference	Leaky gut allows LPS to enter the liver via the portal vein	Inhibition of FXR signaling Upregulation of CYP7A1 expression	Decreased abundance of butyrate-producing bacteria
Impact on Intestine	Intestinal mucosal barrier damage Increased permeability	Bile acids reflux Further damage to intestinal mucosa	Insufficient energy for intestinal epithelium Decreased barrier stability
Impact on Liver	Activation of TLR4/NF-κB pathway Release of inflammatory cytokines	Disruption of bile acids pool	Increased inflammatory cytokines transported via the portal vein Aggravated inflammation
Axis Effect	Amplifies hepatic inflammatory injury	Forms a closed-loop vicious cycle of intestinal and hepatic injury	Persistent damage and chronic deterioration of the intestinal barrier

Note: LPS, lipopolysaccharide; FXR, farnesoid X receptor; SCFA, short-chain fatty acid; CYP7A1, cholesterol 7α-hydroxylase; TLR4, Toll-like receptor 4; NF-κB, nuclear factor kappa-B.

Arsenic is first absorbed through the intestine, directly damaging the intestinal mucosa and increasing intestinal permeability. Subsequently, arsenic entering the bloodstream is transported to the liver via the circulation. Since the liver is responsible for arsenic methylation metabolism and is enriched with various metabolic enzymes, arsenic then accumulates substantially in liver tissue, gradually inducing hepatotoxicity (Prakash et al., 2022). Meanwhile, arsenic can also induce local inflammation in the intestine and liver separately: arsenic activates the NF‑κB pathway in intestinal epithelial cells, promoting the massive release of pro‑inflammatory cytokines such as TNF‑α and IL‑1β; in turn, the intestinal inflammatory microenvironment downregulates the expression of tight junction proteins including CLDN1 and ZO‑1, further deteriorating intestinal permeability and inducing leaky gut (Ibrahim et al., 2022; Li, 2024).

After the intestinal barrier is damaged, LPS that was originally confined to the intestinal lumen enters the liver via the portal vein, becoming an important cause of liver injury secondary to the direct toxicity of arsenic. Immediately thereafter, Kupffer cells recognize LPS through TLR4 and re‑activate the NF‑κB inflammatory pathway, resulting in hepatocyte damage and hepatic steatosis (Hsu and Schnabl, 2023; Rajapakse et al., 2023). As the direct toxicity of arsenic combines with persistent inflammation, hepatic stellate cells are co-activated, and the intracellular TGF-β1/Smad2 pathway is stimulated, prompting the massive synthesis of α-SMA and type I collagen. Hepatic stellate cells then transdifferentiate into myofibroblasts, eventually inducing irreversible liver fibrosis. Moreover, arsenic disrupts the normal pattern of bile acid secretion by the liver, and the abnormally increased bile acids reflux into the intestine and cause damage, thereby establishing a closed‑loop vicious cycle that perpetuates intestinal‑hepatic injury (Li et al., 2025).

Arsenic also alters the gut microbiota structure, indirectly disrupting the normal intercommunication between the intestine and liver. Existing poultry studies have shown that arsenic exposure reduces the α‑diversity of the gut microbiota; beneficial bacteria capable of producing butyrate, such as *Ruminococcaceae*, decrease substantially in number, while arsenic‑tolerant and pro‑inflammatory taxa like *Enterobacteriaceae* proliferate (Y. Yang et al., 2023). This dysbiosis brings about two layers of cascading negative effects: on one hand, the supply of SCFAs such as butyrate is insufficient, depriving the intestinal epithelium of energy support and continuously compromising intestinal barrier stability; on the other hand, the capacity of gut bacteria to modify bile acids is diminished, resulting in obstruction of the FXR‑mediated negative feedback regulation of bile acids on the liver, thereby interrupting the metabolic signal exchange between the intestine and liver (Li, 2024). In addition, the gut microbiota may also alter arsenic speciation through redox reactions and interact bidirectionally with arsenic; however, the specific mechanisms in poultry remain to be further elucidated (Yahong and Weihua, 2025) (Fig. 2).

**Figure 2:**
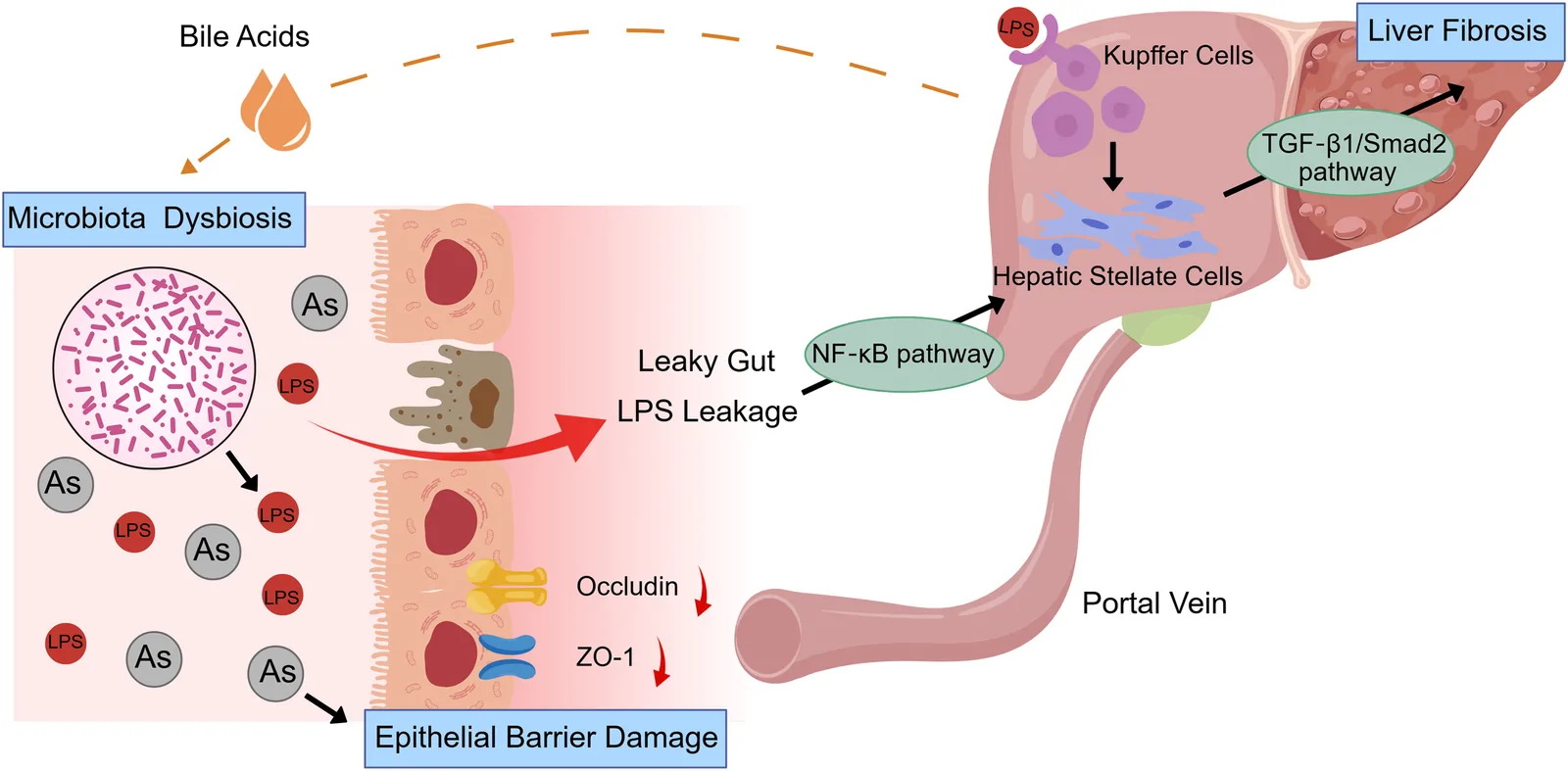
Arsenic‑induced gut microbiota dysbiosis and intestinal epithelial barrier damage trigger LPS leakage, which activates hepatic inflammatory and TGF‑β1/Smad2 signalling cascades to promote liver fibrosis via the gut‑liver axis.

## Prevention and treatment of arsenic poisoning in poultry

The prevention and control of arsenic poisoning in poultry can be approached through three major strategies: promoting arsenic metabolism and excretion, protecting the intestinal and hepatic barriers, and counteracting oxidative stress and inflammation (Table 2). Accelerating arsenic methylation is a direct approach to reducing toxicity (Coryell et al., 2018). The stability of the intestinal barrier and gut microbiota is the key to blocking the cascading damage caused by arsenic along the gut-microbiota-liver axis. *Dendrobium officinale* polysaccharides and *Lactobacillus salivarius*, among others, can upregulate the expression of tight junction proteins such as Claudin‑1 and ZO‑1, repairing the structural damage to the intestine caused by arsenic; at the same time, they can optimize the microbial composition, enrich beneficial bacteria, and enhance the synthesis of SCFAs. Sufficient SCFAs can provide energy for intestinal epithelial cells and suppress intestinal inflammation, thereby reducing the risk of leaky gut from both the physical barrier and intestinal microecological aspects, and decreasing the translocation of LPS and inflammatory cytokines to the liver (Liu et al., 2025; J. Yang et al., 2023).

**Table 2:** Intervention strategies against arsenic toxicity in poultry.

Intervention Mechanism	As-induced Pathological Process	Representative Interventions	Specific Effects	References
Detoxification metabolism	Inorganic arsenic accumulation	Zinc gut commensal bacteria (e.g., *Faecalibacterium*)	Promote the conversion of inorganic arsenic to DMA Microbiota-mediated arsenic biotransformation	Coryell et al., 2018
Barrier repair and microbiota remodeling	LPS translocation to liver causes hepatic injury	*Dendrobium officinale* polysaccharides *Lactobacillus salivariu*s	Upregulate Claudin-1/ZO-1 Enrich beneficial bacteria Enhance SCFA production	Liu et al., 2025 J. Yang et al., 2023
Antioxidant and anti-inflammatory defense	ROS, NF-κB, cascade inflammation	Selenium Vitamin E Phycocyanin	Enhance GSH/GPx Scavenge free radicals Inhibit NF-κB	Ren, et al., 2021 Mashkoor et al., 2023 Talha et al., 2022

Note: DMA, dimethylarsinic acid; LPS, lipopolysaccharide; SCFA, short-chain fatty acid; ROS, reactive oxygen species; GSH-Px, glutathione peroxidase.

Various nutrients can alleviate the systemic toxicity of arsenic through antioxidant effects and inhibition of inflammatory pathways. Dietary selenium (5-10 mg/kg) participates in the synthesis of selenoproteins such as GSH‑Px, reinforcing the GSH‑based antioxidant system, scavenging reactive oxygen species, inhibiting Caspase‑3 activation, and reducing apoptosis; however, selenium at 15 mg/kg exacerbates arsenic toxicity (Ren, Wu, et al., 2021). Vitamin E and spirulina phycocyanin can directly scavenge free radicals, increase the activities of SOD and CAT, decrease MDA levels, and alleviate lipid peroxidation damage (Mashkoor et al., 2023; Talha et al., 2022). A feeding trial showed that combined supplementation with 150 mg/kg vitamin E and 5% bentonite can ameliorate growth retardation in arsenic‑exposed broilers, increase body weight, optimize the feed conversion ratio, alleviate systemic oxidative stress, and reduce pathological damage to organs such as the liver and kidneys (Mashkoor et al., 2023).

From a therapeutic mechanistic perspective, combined supplementation with probiotics, selenium/vitamin E, and methyl donors may theoretically produce synergistic effects; however, factorial-design studies that quantitatively distinguish additive, synergistic, and antagonistic effects of such combinations in poultry are currently lacking, and dose optimization and practical feasibility of combined regimens remain to be established.

## Summary and discussion

This review systematically delineates the dual hazards of arsenic exposure in poultry under production conditions: excessive arsenic accumulation not only suppresses growth, impairs immunity, and leaves residues in meat and eggs, posing a public health concern through the food chain, but also targets the intestine and liver separately. In the intestine, arsenic activates the NF-κB pathway and NLRP3 inflammasome to induce epithelial apoptosis and pyroptosis, downregulates tight junction proteins leading to leaky gut, and reduces butyrate-producing beneficial bacteria while enriching pro-inflammatory taxa. In the liver, arsenic interferes with energy metabolism, depletes the antioxidant system, and triggers multiple programmed cell death pathways including ferroptosis and PANoptosis, progressively leading to steatosis and fibrosis. The gut-microbiota-liver axis links and amplifies injury in both organs: gut-derived LPS aggravates hepatic inflammation via the portal vein, excessive bile acid reflux worsens intestinal damage, and dysbiosis further alters arsenic biotransformation, forming a vicious cycle.

However, current studies remain limited in several respects: poultry-specific mechanistic evidence is still scarce and many inferences are extrapolated from mammals; dose-time-effect relationships are poorly defined because most studies use single doses and short exposure periods; comparative toxicity data across arsenic species in the gut-liver axis are insufficient; multi-omics integration is rare; and the synergistic or antagonistic effects of combined interventions remain largely unexplored.

From a therapeutic perspective, a combined scheme integrating probiotics, selenium/vitamin E, and methyl donors is mechanistically plausible and may reduce intestinal permeability, LPS translocation, and hepatic oxidative injury while improving growth performance. However, optimal doses and ratios require systematic optimization, and factorial-design trials are needed to distinguish additive, synergistic, and antagonistic effects in poultry. Future research should therefore prioritize poultry-specific mechanisms, multi-dose and multi-time-point exposure designs, comparative toxicity across arsenic species, multi-omics integration, and field validation of combined interventions.

## Author contributions

All authors meet the four criteria for authorship as defined by Poultry Science: (1) substantial contributions to the conception or design of the work, or the acquisition, analysis, or interpretation of data; (2) drafting the manuscript or revising it critically for important intellectual content; (3) final approval of the version to be published; and (4) agreement to be accountable for all aspects of the work.

Specific contributions of each author are summarized below:

• Xinying Zhou: Conducted the literature search and data analysis, drafted the original manuscript, and prepared the figures. Approved the final version and agrees to be accountable for all aspects of the work.
• Fuhao Chen: Conceived and designed the review, performed formal analysis, and critically revised the manuscript. Approved the final version and agrees to be accountable for all aspects of the work.
• Qingjia Lin: Conducted the literature search, curated data, provided resources, and validated the results. Approved the final version and agrees to be accountable for all aspects of the work.
• Xinyu Zhang: Conducted the literature search, developed methodology, performed software analysis, and curated data. Approved the final version and agrees to be accountable for all aspects of the work.
• Ting Zhao: Conducted the literature search, curated data, performed formal analysis, and validated the results. Approved the final version and agrees to be accountable for all aspects of the work.
• Falong Yan: Provided resources, supervised the work, critically revised the manuscript, and acquired funding. Approved the final version and agrees to be accountable for all aspects of the work.
• Kai Zhang: Developed methodology, critically revised the manuscript, and performed software analysis. Approved the final version and agrees to be accountable for all aspects of the work.
• Haifeng Liu: Conceived the review, supervised the work, acquired funding, and administered the project. Approved the final version and agrees to be accountable for all aspects of the work.
• Shiwen Xu: Conducted the literature search, prepared figures, validated the results, and curated data. Approved the final version and agrees to be accountable for all aspects of the work.
• Dechun Chen: Conceived and designed the review, conducted the literature search, critically revised the manuscript, administered the project, supervised the work, and acquired funding. Approved the final version and agrees to be accountable for all aspects of the work.

## Funding

This work was supported by 10.13039/501100001809National Natural Science Foundation of China (No. 32673953, 32273052), 10.13039/501100018542Natural Science Foundation of Sichuan Province (No. 2026NSFSC0168), Fengdu County Technological Innovation and Application Development Project (No. FDkJCX202501).

## Disclosures

The authors declare that they have no known competing financial interests or personal relationships that could have appeared to influence the work reported in this paper.
